# 1-Benzyl-4-chloro­indoline-2,3-dione

**DOI:** 10.1107/S1600536811051816

**Published:** 2011-12-07

**Authors:** De Cai Wang, Bo Rong Leng, Gui Bin Wang, Ping Wei, Ping Kai Ou-yang

**Affiliations:** a Sate Key Laboratory of Materials-Oriented Chemcial Engineering, College of Life Science and Pharmaceutical Engineering, Nanjing University of Technology, Xinmofan Road No. 5 Nanjing, Nanjing 210009, People’s Republic of China; b PRC DAYAOWAN Administration for Entry & Exit Inspection and Quarantine, Haiqingdao Foreign Area Development Zone, Dalian 116610, Liaoning Province, People’s Republic of China

## Abstract

There are two independent mol­ecules in the asymmetric unit of the title compound, C_15_H_10_ClNO_2_, which differ in the dihedral angles between the mean planes of the phenyl ring and the 4-chloro­indoline-2,3-dione ring system [59.48 (9) and 79.0 (1)°]. In the crystal, mol­ecules are linked through C—H⋯O hydrogen bonds, forming polymeric chains in [100].

## Related literature

For the preparation, see: Bouhfid *et al.* (2005 ▶). For a related structure and background to isatin derivatives, see: Liu *et al.* (2011 ▶). For reference bond-length data, see: Allen *et al.* (1987 ▶).
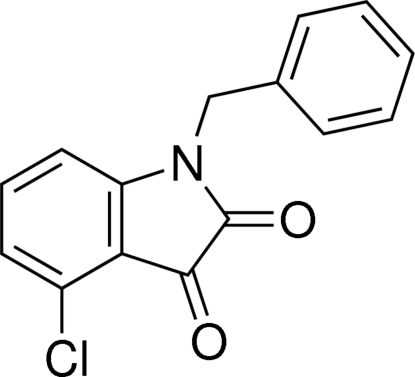


## Experimental

### 

#### Crystal data

C_15_H_10_ClNO_2_
*M* *_r_* = 271.69
Orthorhombic, 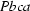

*a* = 22.864 (5) Å
*b* = 16.600 (3) Å
*c* = 13.335 (3) Å
*V* = 5061.2 (18) Å^3^
*Z* = 16
Mo *K*α radiation
μ = 0.30 mm^−1^
*T* = 293 K
0.30 × 0.20 × 0.10 mm

#### Data collection

Enraf–Nonius CAD-4 diffractometer
Absorption correction: ψ scan (North *et al.*, 1968 ▶) *T*_min_ = 0.916, *T*_max_ = 0.971
4623 measured reflections
4623 independent reflections
1929 reflections with *I* > 2σ(*I*)
3 standard reflections every 200 reflections  intensity decay: 1%

#### Refinement

*R*[*F* ^2^ > 2σ(*F* ^2^)] = 0.064
*wR*(*F* ^2^) = 0.117
*S* = 1.00
4623 reflections
343 parameters
H-atom parameters constrained
Δρ_max_ = 0.18 e Å^−3^
Δρ_min_ = −0.20 e Å^−3^

### 

Data collection: *CAD-4 EXPRESS* (Enraf–Nonius, 1994 ▶); cell refinement: *CAD-4 EXPRESS*; data reduction: *XCAD4* (Harms & Wocadlo,1995 ▶); program(s) used to solve structure: *SHELXS97* (Sheldrick, 2008 ▶); program(s) used to refine structure: *SHELXL97* (Sheldrick, 2008 ▶); molecular graphics: *SHELXTL* (Sheldrick, 2008 ▶); software used to prepare material for publication: *PLATON* (Spek, 2009 ▶).

## Supplementary Material

Crystal structure: contains datablock(s) global, I. DOI: 10.1107/S1600536811051816/hb6540sup1.cif

Structure factors: contains datablock(s) I. DOI: 10.1107/S1600536811051816/hb6540Isup2.hkl

Supplementary material file. DOI: 10.1107/S1600536811051816/hb6540Isup3.cml

Additional supplementary materials:  crystallographic information; 3D view; checkCIF report

## Figures and Tables

**Table 1:** Hydrogen-bond geometry (Å, °)

*D*—H⋯*A*	*D*—H	H⋯*A*	*D*⋯*A*	*D*—H⋯*A*
C3—H3*A*⋯O1^i^	0.93	2.58	3.228 (4)	127
C4—H4*A*⋯O2^i^	0.93	2.56	3.473 (4)	167
C18—H18*A*⋯O3^ii^	0.93	2.40	3.329 (4)	173
C19—H19*A*⋯O4^ii^	0.93	2.60	3.382 (6)	142
C26—H26*A*⋯O2^iii^	0.93	2.59	3.350 (4)	140
C29—H29*A*⋯O1^iv^	0.93	2.59	3.505 (5)	169

Symmetry codes: (i) 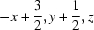; (ii) 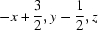; (iii) 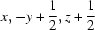; (iv) 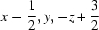.

**Fig. 1.**
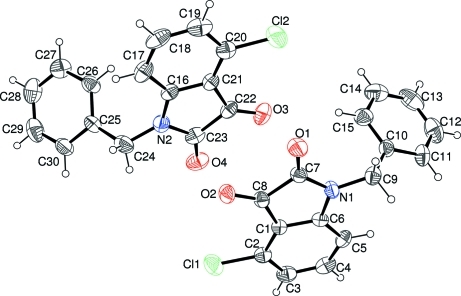
The molecular structure of (I), showing displacement ellipsoids at the 30% probability level.

**Fig. 2.**
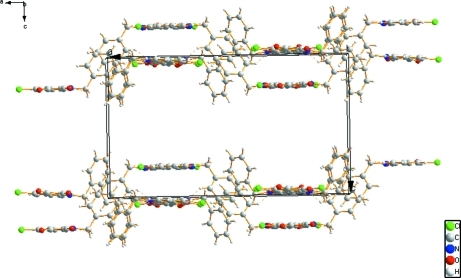
A packing diagram of (I). The intermolecular hydrogen bonds are shown as dashed lines.

## supplementary crystallographic information

### Comment

As a part of our studies into the synthesis and structures of isatin derivatives (Liu *et al.*, 2011), the title compound (I) was synthesized (Bouhfid *et al.*2005) and its structure is now reported.

The title compound crystallized with two independent molecules (A & B) in the asymmetric unit (Fig. 1). They differ significantly in conformation, as may be seen from the dihedral angle in the mean planes of the benzene and 4-chloroindoline-2,3-dione. For molecule A,the dihedral angle between the mean planes of the benzene and 4-chloroindoline-2,3-dione is 59.481 (88)°,while the corresponding dihedral angle is 79.028 (114)° in molecule B. The bond lengths (Allen *et al.*, 1987) and bond angles are otherwise within normal ranges.

In the crystal structure, intermolecular and intramolecular C—H···O hydrogen bonding interactions (Table 1) link the molecules into a polymeric chain extended along the *a* axis (Fig. 2).

### Experimental

4-chloroisatin (1.81 g, 0.01 mol) was reacted with benzyl bromide (0.02 mol) in the presence of K_2_CO_3_ (2.76 g, 0.02 mol) and tetrabutylammonium bromide (0.32 g, 0.001 mol) in DMF (60 ml). After 12 h stirring at rt, the precipitate was removed by filtration and purified by recrystallization from ethanol (m.p. 165.8–166.5 °C; yield 70%). Yellow blocks of the title compound were obtained by slow evaporation of an ethanol solution at room temperature.

### Refinement

All H atoms were placed geometrically (C—H = 0.93–0.96 Å) and refined as riding with *U*_iso_(H) = 1.2*U*_eq_(carrier) or 1.5*U*_eq_(methyl carrier).

### Crystal data

**Table:**

C_15_H_10_ClNO_2_	*F*(000) = 2240
*M**_r_* = 271.69	*D*_x_ = 1.426 Mg m^−^^3^
Orthorhombic, *P**b**c**a*	Mo *K*α radiation, λ = 0.71073 Å
Hall symbol: -P 2ac 2ab	Cell parameters from 25 reflections
*a* = 22.864 (5) Å	θ = 9–13°
*b* = 16.600 (3) Å	µ = 0.30 mm^−^^1^
*c* = 13.335 (3) Å	*T* = 293 K
*V* = 5061.2 (18) Å^3^	Block, yellow
*Z* = 16	0.30 × 0.20 × 0.10 mm

### Data collection

**Table:**

Enraf–Nonius CAD-4 diffractometer	1929 reflections with *I* > 2σ(*I*)
Radiation source: fine-focus sealed tube	*R*_int_ = 0.000
graphite	θ_max_ = 25.4°, θ_min_ = 1.8°
ω/2θ scans	*h* = 0→27
Absorption correction: ψ scan (North *et al.*, 1968)	*k* = 0→20
*T*_min_ = 0.916, *T*_max_ = 0.971	*l* = 0→16
4623 measured reflections	3 standard reflections every 200 reflections
4623 independent reflections	intensity decay: 1%

### Refinement

**Table:**

Refinement on *F*^2^	Primary atom site location: structure-invariant direct methods
Least-squares matrix: full	Secondary atom site location: difference Fourier map
*R*[*F*^2^ > 2σ(*F*^2^)] = 0.064	Hydrogen site location: inferred from neighbouring sites
*wR*(*F*^2^) = 0.117	H-atom parameters constrained
*S* = 1.00	*w* = 1/[σ^2^(*F*_o_^2^) + (0.032*P*)^2^] where *P* = (*F*_o_^2^ + 2*F*_c_^2^)/3
4623 reflections	(Δ/σ)_max_ = 0.001
343 parameters	Δρ_max_ = 0.18 e Å^−^^3^
0 restraints	Δρ_min_ = −0.20 e Å^−^^3^

### Special details

**Table:**

Geometry. All e.s.d.'s (except the e.s.d. in the dihedral angle between two l.s. planes) are estimated using the full covariance matrix. The cell e.s.d.'s are taken into account individually in the estimation of e.s.d.'s in distances, angles and torsion angles; correlations between e.s.d.'s in cell parameters are only used when they are defined by crystal symmetry. An approximate (isotropic) treatment of cell e.s.d.'s is used for estimating e.s.d.'s involving l.s. planes.
Refinement. Refinement of *F*^2^ against ALL reflections. The weighted *R*-factor *wR* and goodness of fit *S* are based on *F*^2^, conventional *R*-factors *R* are based on *F*, with *F* set to zero for negative *F*^2^. The threshold expression of *F*^2^ > σ(*F*^2^) is used only for calculating *R*-factors(gt) *etc*. and is not relevant to the choice of reflections for refinement. *R*-factors based on *F*^2^ are statistically about twice as large as those based on *F*, and *R*- factors based on ALL data will be even larger.

### Fractional atomic coordinates and isotropic or equivalent isotropic displacement parameters (Å^2^)

**Table:**

	*x*	*y*	*z*	*U*_iso_*/*U*_eq_	
Cl1	0.61720 (4)	0.52555 (7)	0.54905 (10)	0.0902 (4)	
C1	0.73389 (14)	0.5069 (2)	0.5352 (3)	0.0438 (9)	
N1	0.83104 (11)	0.47219 (17)	0.5229 (2)	0.0506 (8)	
O1	0.82562 (10)	0.33371 (14)	0.5350 (2)	0.0681 (8)	
O2	0.70097 (10)	0.36781 (14)	0.5538 (2)	0.0628 (8)	
C2	0.68738 (15)	0.5601 (2)	0.5384 (3)	0.0522 (10)	
C3	0.69793 (18)	0.6420 (2)	0.5343 (3)	0.0644 (12)	
H3A	0.6671	0.6785	0.5358	0.077*	
C4	0.7549 (2)	0.6687 (2)	0.5278 (3)	0.0649 (12)	
H4A	0.7617	0.7239	0.5269	0.078*	
C5	0.80188 (17)	0.6178 (2)	0.5228 (3)	0.0540 (11)	
H5A	0.8398	0.6375	0.5173	0.065*	
C6	0.79077 (15)	0.5362 (2)	0.5261 (3)	0.0452 (9)	
C7	0.80405 (15)	0.3997 (2)	0.5335 (3)	0.0490 (10)	
C8	0.73753 (16)	0.4189 (2)	0.5423 (3)	0.0489 (10)	
C9	0.89331 (15)	0.4816 (2)	0.5095 (3)	0.0576 (11)	
H9A	0.9002	0.5207	0.4567	0.069*	
H9B	0.9096	0.4306	0.4876	0.069*	
C10	0.92469 (15)	0.5083 (2)	0.6021 (3)	0.0525 (10)	
C11	0.96343 (16)	0.5718 (2)	0.5974 (3)	0.0721 (13)	
H11A	0.9693	0.5990	0.5373	0.086*	
C12	0.9933 (2)	0.5946 (3)	0.6822 (5)	0.0975 (17)	
H12A	1.0196	0.6373	0.6790	0.117*	
C13	0.9849 (2)	0.5558 (4)	0.7704 (5)	0.1020 (19)	
H13A	1.0054	0.5717	0.8273	0.122*	
C14	0.9457 (2)	0.4926 (3)	0.7757 (4)	0.0911 (15)	
H14A	0.9398	0.4657	0.8360	0.109*	
C15	0.91552 (17)	0.4698 (2)	0.6912 (3)	0.0674 (12)	
H15A	0.8886	0.4279	0.6947	0.081*	
O3	0.78599 (12)	0.39116 (16)	0.7763 (2)	0.0807 (9)	
O4	0.66106 (12)	0.42670 (16)	0.7850 (2)	0.0824 (9)	
N2	0.65413 (13)	0.28816 (19)	0.7866 (2)	0.0596 (9)	
Cl2	0.86828 (5)	0.23348 (8)	0.78394 (10)	0.1009 (5)	
C16	0.69392 (17)	0.2242 (2)	0.7838 (3)	0.0512 (10)	
C17	0.6827 (2)	0.1434 (2)	0.7823 (3)	0.0699 (12)	
H17A	0.6444	0.1245	0.7811	0.084*	
C18	0.7288 (2)	0.0906 (2)	0.7827 (3)	0.0789 (14)	
H18A	0.7214	0.0355	0.7817	0.095*	
C19	0.7861 (2)	0.1170 (3)	0.7845 (3)	0.0767 (14)	
H19A	0.8167	0.0802	0.7859	0.092*	
C20	0.79739 (17)	0.1997 (3)	0.7842 (3)	0.0633 (11)	
C21	0.75154 (15)	0.2526 (2)	0.7840 (3)	0.0506 (10)	
C22	0.74875 (18)	0.3404 (2)	0.7816 (3)	0.0551 (11)	
C23	0.68310 (19)	0.3602 (3)	0.7847 (3)	0.0618 (11)	
C24	0.59128 (16)	0.2818 (3)	0.7776 (3)	0.0758 (13)	
H24A	0.5827	0.2432	0.7252	0.091*	
H24B	0.5764	0.3336	0.7556	0.091*	
C25	0.55801 (16)	0.2574 (2)	0.8702 (3)	0.0587 (11)	
C26	0.58256 (16)	0.2129 (2)	0.9452 (3)	0.0647 (12)	
H26A	0.6223	0.2009	0.9437	0.078*	
C27	0.5485 (2)	0.1861 (3)	1.0228 (3)	0.0774 (13)	
H27A	0.5653	0.1545	1.0727	0.093*	
C28	0.4906 (2)	0.2049 (3)	1.0284 (4)	0.0900 (16)	
H28A	0.4677	0.1864	1.0813	0.108*	
C29	0.46671 (18)	0.2517 (3)	0.9539 (5)	0.0910 (16)	
H29A	0.4275	0.2662	0.9573	0.109*	
C30	0.49984 (18)	0.2771 (2)	0.8754 (3)	0.0736 (13)	
H30A	0.4830	0.3079	0.8249	0.088*	

### Atomic displacement parameters (Å^2^)

**Table:**

	*U*^11^	*U*^22^	*U*^33^	*U*^12^	*U*^13^	*U*^23^
Cl1	0.0555 (7)	0.0927 (9)	0.1224 (10)	0.0131 (6)	0.0004 (7)	0.0070 (8)
C1	0.046 (2)	0.043 (2)	0.042 (2)	0.0025 (19)	0.001 (2)	−0.0029 (19)
N1	0.0396 (17)	0.0442 (18)	0.068 (2)	−0.0015 (15)	0.0006 (17)	−0.0029 (18)
O1	0.0652 (17)	0.0403 (16)	0.099 (2)	0.0090 (14)	−0.0063 (16)	−0.0039 (16)
O2	0.0551 (16)	0.0472 (16)	0.086 (2)	−0.0117 (14)	−0.0087 (16)	−0.0008 (15)
C2	0.059 (2)	0.048 (2)	0.050 (3)	0.007 (2)	0.001 (2)	−0.002 (2)
C3	0.077 (3)	0.052 (3)	0.064 (3)	0.018 (2)	0.000 (3)	0.001 (2)
C4	0.096 (3)	0.048 (3)	0.051 (3)	0.002 (3)	0.001 (3)	−0.002 (2)
C5	0.066 (3)	0.040 (2)	0.056 (3)	−0.008 (2)	0.007 (2)	0.002 (2)
C6	0.050 (2)	0.043 (2)	0.043 (2)	0.0059 (19)	−0.0061 (19)	−0.001 (2)
C7	0.049 (2)	0.043 (2)	0.056 (3)	−0.001 (2)	−0.009 (2)	−0.002 (2)
C8	0.050 (2)	0.046 (3)	0.050 (3)	−0.007 (2)	−0.010 (2)	−0.005 (2)
C9	0.055 (2)	0.054 (3)	0.063 (3)	−0.002 (2)	0.010 (2)	0.000 (2)
C10	0.045 (2)	0.055 (3)	0.058 (3)	0.0001 (19)	0.007 (2)	0.001 (2)
C11	0.056 (3)	0.071 (3)	0.089 (4)	−0.019 (2)	0.008 (3)	−0.004 (3)
C12	0.066 (3)	0.097 (4)	0.129 (5)	−0.017 (3)	−0.007 (4)	−0.031 (4)
C13	0.081 (4)	0.126 (5)	0.099 (5)	0.016 (4)	−0.041 (4)	−0.041 (4)
C14	0.100 (4)	0.100 (4)	0.073 (4)	0.022 (3)	−0.012 (3)	0.006 (3)
C15	0.070 (3)	0.056 (3)	0.076 (3)	0.004 (2)	−0.006 (3)	0.002 (3)
O3	0.090 (2)	0.0580 (18)	0.095 (2)	−0.0212 (17)	0.0114 (19)	0.0033 (18)
O4	0.107 (2)	0.0579 (18)	0.082 (2)	0.0191 (17)	0.0051 (19)	0.0061 (18)
N2	0.057 (2)	0.059 (2)	0.063 (2)	−0.0011 (18)	0.003 (2)	0.003 (2)
Cl2	0.0643 (7)	0.1260 (11)	0.1124 (11)	0.0158 (8)	0.0084 (8)	0.0130 (9)
C16	0.068 (3)	0.042 (2)	0.044 (2)	−0.002 (2)	0.000 (2)	−0.001 (2)
C17	0.097 (3)	0.053 (3)	0.060 (3)	−0.018 (3)	0.010 (3)	−0.008 (3)
C18	0.137 (5)	0.039 (3)	0.060 (3)	0.003 (3)	0.008 (3)	0.000 (2)
C19	0.105 (4)	0.070 (3)	0.055 (3)	0.031 (3)	0.007 (3)	−0.002 (3)
C20	0.072 (3)	0.066 (3)	0.052 (3)	0.011 (2)	0.009 (2)	0.001 (2)
C21	0.061 (2)	0.044 (2)	0.047 (2)	−0.004 (2)	0.003 (2)	−0.0032 (19)
C22	0.073 (3)	0.048 (3)	0.044 (2)	−0.007 (2)	0.006 (2)	0.000 (2)
C23	0.084 (3)	0.052 (3)	0.049 (3)	0.008 (3)	0.007 (2)	0.000 (3)
C24	0.064 (3)	0.099 (3)	0.065 (3)	−0.002 (2)	−0.018 (3)	0.006 (3)
C25	0.047 (2)	0.069 (3)	0.060 (3)	0.002 (2)	−0.010 (2)	−0.005 (2)
C26	0.055 (3)	0.088 (3)	0.051 (3)	0.007 (2)	−0.002 (2)	−0.003 (3)
C27	0.087 (3)	0.082 (3)	0.063 (3)	0.012 (3)	0.012 (3)	−0.001 (3)
C28	0.090 (4)	0.092 (4)	0.088 (4)	−0.016 (3)	0.035 (3)	−0.023 (3)
C29	0.055 (3)	0.091 (4)	0.128 (5)	−0.001 (3)	0.011 (4)	−0.024 (4)
C30	0.057 (3)	0.074 (3)	0.090 (4)	0.002 (2)	−0.021 (3)	0.002 (3)

### Geometric parameters (Å, °)

**Table:**

Cl1—C2	1.710 (4)	O3—C22	1.200 (4)
C1—C2	1.383 (4)	O4—C23	1.213 (4)
C1—C6	1.394 (4)	N2—C23	1.368 (4)
C1—C8	1.465 (4)	N2—C16	1.399 (4)
N1—C7	1.359 (4)	N2—C24	1.446 (4)
N1—C6	1.407 (4)	Cl2—C20	1.715 (4)
N1—C9	1.444 (4)	C16—C17	1.366 (4)
O1—C7	1.202 (4)	C16—C21	1.399 (4)
O2—C8	1.201 (4)	C17—C18	1.372 (5)
C2—C3	1.382 (4)	C17—H17A	0.9300
C3—C4	1.378 (5)	C18—C19	1.381 (5)
C3—H3A	0.9300	C18—H18A	0.9300
C4—C5	1.369 (5)	C19—C20	1.397 (5)
C4—H4A	0.9300	C19—H19A	0.9300
C5—C6	1.379 (4)	C20—C21	1.368 (5)
C5—H5A	0.9300	C21—C22	1.459 (5)
C7—C8	1.558 (4)	C22—C23	1.537 (5)
C9—C10	1.496 (5)	C24—C25	1.506 (5)
C9—H9A	0.9700	C24—H24A	0.9700
C9—H9B	0.9700	C24—H24B	0.9700
C10—C15	1.365 (5)	C25—C26	1.364 (5)
C10—C11	1.378 (4)	C25—C30	1.371 (5)
C11—C12	1.374 (6)	C26—C27	1.369 (5)
C11—H11A	0.9300	C26—H26A	0.9300
C12—C13	1.354 (6)	C27—C28	1.363 (5)
C12—H12A	0.9300	C27—H27A	0.9300
C13—C14	1.381 (6)	C28—C29	1.374 (6)
C13—H13A	0.9300	C28—H28A	0.9300
C14—C15	1.375 (5)	C29—C30	1.359 (5)
C14—H14A	0.9300	C29—H29A	0.9300
C15—H15A	0.9300	C30—H30A	0.9300
			
C2—C1—C6	119.8 (3)	C23—N2—C16	110.4 (3)
C2—C1—C8	132.7 (3)	C23—N2—C24	122.9 (4)
C6—C1—C8	107.5 (3)	C16—N2—C24	126.1 (3)
C7—N1—C6	111.6 (3)	C17—C16—N2	128.6 (4)
C7—N1—C9	123.8 (3)	C17—C16—C21	120.6 (4)
C6—N1—C9	124.6 (3)	N2—C16—C21	110.9 (3)
C3—C2—C1	119.5 (3)	C16—C17—C18	118.8 (4)
C3—C2—Cl1	119.8 (3)	C16—C17—H17A	120.6
C1—C2—Cl1	120.7 (3)	C18—C17—H17A	120.6
C4—C3—C2	118.9 (4)	C17—C18—C19	121.8 (4)
C4—C3—H3A	120.5	C17—C18—H18A	119.1
C2—C3—H3A	120.5	C19—C18—H18A	119.1
C5—C4—C3	123.1 (4)	C18—C19—C20	119.2 (4)
C5—C4—H4A	118.4	C18—C19—H19A	120.4
C3—C4—H4A	118.4	C20—C19—H19A	120.4
C4—C5—C6	117.4 (4)	C21—C20—C19	119.3 (4)
C4—C5—H5A	121.3	C21—C20—Cl2	121.0 (3)
C6—C5—H5A	121.3	C19—C20—Cl2	119.7 (4)
C5—C6—C1	121.2 (3)	C20—C21—C16	120.3 (4)
C5—C6—N1	128.4 (3)	C20—C21—C22	132.4 (4)
C1—C6—N1	110.4 (3)	C16—C21—C22	107.2 (3)
O1—C7—N1	128.5 (3)	O3—C22—C21	132.2 (4)
O1—C7—C8	125.9 (3)	O3—C22—C23	123.0 (4)
N1—C7—C8	105.6 (3)	C21—C22—C23	104.8 (3)
O2—C8—C1	132.3 (4)	O4—C23—N2	126.5 (4)
O2—C8—C7	123.0 (3)	O4—C23—C22	126.9 (4)
C1—C8—C7	104.8 (3)	N2—C23—C22	106.6 (3)
N1—C9—C10	113.7 (3)	N2—C24—C25	117.0 (3)
N1—C9—H9A	108.8	N2—C24—H24A	108.1
C10—C9—H9A	108.8	C25—C24—H24A	108.1
N1—C9—H9B	108.8	N2—C24—H24B	108.1
C10—C9—H9B	108.8	C25—C24—H24B	108.1
H9A—C9—H9B	107.7	H24A—C24—H24B	107.3
C15—C10—C11	119.7 (4)	C26—C25—C30	119.4 (4)
C15—C10—C9	120.4 (4)	C26—C25—C24	122.7 (4)
C11—C10—C9	119.8 (4)	C30—C25—C24	117.9 (4)
C12—C11—C10	119.5 (4)	C25—C26—C27	119.8 (4)
C12—C11—H11A	120.2	C25—C26—H26A	120.1
C10—C11—H11A	120.2	C27—C26—H26A	120.1
C13—C12—C11	120.8 (5)	C28—C27—C26	121.3 (5)
C13—C12—H12A	119.6	C28—C27—H27A	119.4
C11—C12—H12A	119.6	C26—C27—H27A	119.4
C12—C13—C14	119.8 (5)	C27—C28—C29	118.4 (5)
C12—C13—H13A	120.1	C27—C28—H28A	120.8
C14—C13—H13A	120.1	C29—C28—H28A	120.8
C15—C14—C13	119.5 (5)	C30—C29—C28	120.7 (4)
C15—C14—H14A	120.2	C30—C29—H29A	119.6
C13—C14—H14A	120.2	C28—C29—H29A	119.6
C10—C15—C14	120.5 (4)	C29—C30—C25	120.4 (4)
C10—C15—H15A	119.8	C29—C30—H30A	119.8
C14—C15—H15A	119.8	C25—C30—H30A	119.8
			
C6—C1—C2—C3	−1.2 (6)	C23—N2—C16—C17	−178.5 (4)
C8—C1—C2—C3	177.0 (4)	C24—N2—C16—C17	−7.2 (6)
C6—C1—C2—Cl1	179.7 (3)	C23—N2—C16—C21	2.4 (5)
C8—C1—C2—Cl1	−2.1 (6)	C24—N2—C16—C21	173.7 (4)
C1—C2—C3—C4	−0.5 (6)	N2—C16—C17—C18	−178.0 (4)
Cl1—C2—C3—C4	178.6 (3)	C21—C16—C17—C18	1.1 (6)
C2—C3—C4—C5	1.7 (6)	C16—C17—C18—C19	0.0 (7)
C3—C4—C5—C6	−1.1 (6)	C17—C18—C19—C20	−1.1 (7)
C4—C5—C6—C1	−0.7 (6)	C18—C19—C20—C21	1.2 (7)
C4—C5—C6—N1	−179.5 (3)	C18—C19—C20—Cl2	−178.7 (4)
C2—C1—C6—C5	1.8 (6)	C19—C20—C21—C16	−0.1 (6)
C8—C1—C6—C5	−176.8 (3)	Cl2—C20—C21—C16	179.7 (3)
C2—C1—C6—N1	−179.2 (3)	C19—C20—C21—C22	−178.6 (4)
C8—C1—C6—N1	2.2 (4)	Cl2—C20—C21—C22	1.3 (7)
C7—N1—C6—C5	176.6 (4)	C17—C16—C21—C20	−1.0 (6)
C9—N1—C6—C5	−3.8 (6)	N2—C16—C21—C20	178.2 (4)
C7—N1—C6—C1	−2.4 (4)	C17—C16—C21—C22	177.8 (3)
C9—N1—C6—C1	177.2 (3)	N2—C16—C21—C22	−3.0 (5)
C6—N1—C7—O1	−179.3 (4)	C20—C21—C22—O3	2.6 (8)
C9—N1—C7—O1	1.1 (6)	C16—C21—C22—O3	−176.0 (4)
C6—N1—C7—C8	1.4 (4)	C20—C21—C22—C23	−179.0 (4)
C9—N1—C7—C8	−178.2 (3)	C16—C21—C22—C23	2.4 (4)
C2—C1—C8—O2	−0.8 (8)	C16—N2—C23—O4	179.0 (4)
C6—C1—C8—O2	177.5 (4)	C24—N2—C23—O4	7.4 (7)
C2—C1—C8—C7	−179.6 (4)	C16—N2—C23—C22	−0.8 (4)
C6—C1—C8—C7	−1.3 (4)	C24—N2—C23—C22	−172.4 (3)
O1—C7—C8—O2	1.7 (6)	O3—C22—C23—O4	−2.2 (7)
N1—C7—C8—O2	−179.0 (3)	C21—C22—C23—O4	179.2 (4)
O1—C7—C8—C1	−179.3 (4)	O3—C22—C23—N2	177.6 (4)
N1—C7—C8—C1	−0.1 (4)	C21—C22—C23—N2	−1.0 (4)
C7—N1—C9—C10	−102.9 (4)	C23—N2—C24—C25	−109.5 (4)
C6—N1—C9—C10	77.5 (4)	C16—N2—C24—C25	80.3 (5)
N1—C9—C10—C15	49.4 (5)	N2—C24—C25—C26	−25.9 (6)
N1—C9—C10—C11	−131.1 (3)	N2—C24—C25—C30	158.0 (4)
C15—C10—C11—C12	1.2 (6)	C30—C25—C26—C27	2.3 (6)
C9—C10—C11—C12	−178.3 (4)	C24—C25—C26—C27	−173.8 (4)
C10—C11—C12—C13	−0.3 (7)	C25—C26—C27—C28	−1.9 (6)
C11—C12—C13—C14	−0.2 (8)	C26—C27—C28—C29	0.0 (7)
C12—C13—C14—C15	−0.1 (7)	C27—C28—C29—C30	1.5 (7)
C11—C10—C15—C14	−1.6 (6)	C28—C29—C30—C25	−1.1 (7)
C9—C10—C15—C14	177.9 (4)	C26—C25—C30—C29	−0.8 (6)
C13—C14—C15—C10	1.1 (7)	C24—C25—C30—C29	175.5 (4)

### Hydrogen-bond geometry (Å, °)

**Table:**

*D*—H···*A*	*D*—H	H···*A*	*D*···*A*	*D*—H···*A*
C3—H3A···O1^i^	0.93	2.58	3.228 (4)	127
C4—H4A···O2^i^	0.93	2.56	3.473 (4)	167
C18—H18A···O3^ii^	0.93	2.40	3.329 (4)	173
C19—H19A···O4^ii^	0.93	2.60	3.382 (6)	142
C26—H26A···O2^iii^	0.93	2.59	3.350 (4)	140
C29—H29A···O1^iv^	0.93	2.59	3.505 (5)	169

Symmetry codes: (i) −*x*+3/2, *y*+1/2, *z*; (ii) −*x*+3/2, *y*−1/2, *z*; (iii) *x*, −*y*+1/2, *z*+1/2; (iv) *x*−1/2, *y*, −*z*+3/2.
